# On the development of surgical techniques for the treatment of onychocryptosis – Answer^☆^^☆☆^

**DOI:** 10.1016/j.abd.2021.05.003

**Published:** 2021-07-15

**Authors:** Han Ma

**Affiliations:** Department of Dermatology, Guangdong Provincial Key, Laboratory of Biomedical Imaging, Fifth Affiliated Hospital, Sun Yat-sen University, Zhuhai, Guangdong Province, China

Dear Editor,

I am very pleased with the attention given to my article^1^. As mentioned in the table of the correspondence,^2^ there are numerous surgical techniques for onychocryptosis. In the first paragraph of my article, I have cited the literature^3^ to emphasize that all the surgical strategies can be categorized into two main approaches: either narrowing the nail plate or debulking the soft tissues. And I have chosen the first one.

The key point to narrow the nail plate is to destroy the corresponding part of the nail matrix completely. Treatments include surgery, electrocautery, and chemicals, etc. The most assured is the surgical excision. There are clear points and lines of reference in every step of the surgical approach I proposed. In the discussion, I emphasized that Step 4 is the most important procedure to avoid recurrence. I was very careful to cut off all the tissue around the corresponding part of the nail matrix in all my 67 patients. And there are still two suggestions: 1) to see the white phalanx; 2) to perform a little wedge-shaped resection. As a result, I am very confident to guarantee low recurrence or even no recurrence after surgery.

I have propagated my technique in more than fifteen hospitals in southern China. Most dermatologists only need to observe and listen once to achieve results similar to mine. Even so, with the established method, there may be a few differences in the final recurrence rate among different doctors.

## Financial support

This was supported by Zhuhai Science and Technology Plan Medical and Health Project (ZH2202200003HJL).

## Author contributions

Han Ma: Approval of the final version of the manuscript; design and planning of the study; drafting and editing of the manuscript; collection, analysis, and interpretation of data; effective participation in research orientation; intellectual participation in the propaedeutic and/or therapeutic conduct of the studied cases; critical review of the literature; critical review of the manuscript.

## Conflicts of interest

None declared.
